# Callus Induction in *Ludwigia octovalvis*: Chemical Characterization and Potential Pharmacological Applications

**DOI:** 10.3390/plants15172590

**Published:** 2026-08-25

**Authors:** Stephany Abigail Tadeo-Cuenca, Silvia Marquina-Bahena, Elizabeth Negrete-León, Juan José Acevedo-Fernández, María Crystal Columba-Palomares, Araceli Guerrero-Alonso, Francisco Cruz-Sosa, Mariana Sánchez-Ramos

**Affiliations:** 1 Department of Biotechnology, Metropolitan Autonomous University-Iztapalapa Campus, Av. Ferrocarril de San Rafael Atlixco 186, Col. Leyes de Reforma 1ª. Sección, Alcaldía Iztapalapa, Mexico City 09310, Mexico; cbs2223801823@izt.uam.mx; 2 Chemical Research Center-IICBA, Autonomous University of the State of Morelos, Av. Universidad 1001, Col. Chamilpa, Cuernavaca 62209, Morelos, Mexico; smarquina@uaem.mx (S.M.-B.); araceli.guerreroa@uaem.edu.mx (A.G.-A.); 3 Electrophysiology and Pharmacological Bioevaluation Laboratory, Faculty of Medicine, Autonomous University of the State of Morelos, Leñeros S/N, Los Volcanes, Cuernavaca 62350, Morelos, Mexico; elizabeth.negrete@uaem.mx (E.N.-L.); juan.acevedo@uaem.mx (J.J.A.-F.); 4 Faculty of Pharmacy, Autonomous University of the State of Morelos, Av. Universidad 1001, Col. Chamilpa, Cuernavaca 62209, Morelos, Mexico; cpmc_ff@uaem.mx

**Keywords:** *Ludwigia octovalvis*, callogenesis, antimicrobial, wound healing

## Abstract

Antimicrobial resistance has intensified the search for novel antimicrobial and wound-healing agents. *Ludwigia octovalvis* (Jacq.) P.H. Raven is traditionally used to treat infections, inflammation, and skin disorders, although its in vitro biotechnological potential remains largely unexplored. This study reports the first successful establishment of *L. octovalvis* callus cultures, their characterization by gas chromatography-mass spectrometry (GC-MS), and the evaluation of their antimicrobial and wound-healing activities. Friable calluses were induced from leaf and node explants of axenic seedlings using combinations of 6-benzylaminopurine (BAP), kinetin (KIN), 2,4-dichlorophenoxyacetic acid (2,4-D), and α-naphthaleneacetic acid (NAA). Optimal callus induction was achieved in leaf explants cultured on full-strength Murashige and Skoog (MS) medium supplemented with BAP (4.44 µM) and 2,4-D (0.45 µM). After six months of subculture, the calluses showed morphological uniformity and stable biomass production. Growth kinetics followed a specific growth rate of 0.047 d^−1^ and a doubling time of 14.54 days. GC-MS analysis identified fatty acids and phytosterols as the main components. The ethyl acetate extract significantly improved wound healing in vivo, while the methanolic extract exhibited antibacterial activity against *Staphylococcus aureus* and methicillin-resistant *S. aureus* (MRSA). These findings demonstrate that *L. octovalvis* callus cultures constitute a sustainable source of bioactive compounds with promising therapeutic potential.

## 1. Introduction

Indiscriminate, inappropriate, and prolonged use of antibiotics in human medicine, veterinary medicine, and the agro-industry constitutes the main epidemiological factor and the main anthropogenic force in the emergence, selection, and dissemination of antimicrobial resistance. Recent projections warn that, if trends continue as they are, this phenomenon could be linked to more than 169 million deaths between 2025 and 2050 [1]. Antimicrobial resistance arises primarily from genetic mechanisms, such as point mutations and horizontal gene transfer, which are favored by the selective pressure exerted by the overuse of antibiotics. This is compounded by well-characterized bacterial resistance strategies, including overexpression of efflux pumps, modification of molecular targets, and enzymatic inactivation of drugs [2,3].

In this context, plant-derived secondary metabolites, including terpenoids, alkaloids, and flavonoids, have emerged as promising sources of bioactive compounds with antimicrobial potential [4,5,6]. These phytochemicals have demonstrated efficacy against multiple cellular targets, including by inhibiting nucleic acid synthesis, altering membrane integrity, inhibiting essential enzymes, and preventing biofilm formation. Their ability to modulate or reverse bacterial resistance mechanisms has also been documented [4,7]. A distinctive feature of these compounds is their low toxicity and a lower likelihood of inducing resistance compared to conventional antibiotics [8].

Interest in natural products has also extended to the field of wound healing. The World Health Organization estimates that approximately 80% of the world’s population uses traditional medicine, primarily based on medicinal plants [9]. Wound healing is a dynamic and highly regulated process that includes phases of hemostasis, inflammation, proliferation, and remodeling, all of which can be compromised by infections, metabolic diseases, aging, or pharmacological treatments [10]. In this context, plant-derived secondary metabolites play a fundamental role in critical processes such as collagen synthesis, cell proliferation, and angiogenesis, promoting tissue repair and functional skin recovery [11].

Medicinal plants, therefore, represent an invaluable source of phytochemicals with therapeutic properties. However, their use is limited by factors such as habitat loss, overexploitation, and low metabolite concentrations in plant tissues [12]. In response to these limitations, plant biotechnology and in vitro culture systems of cells, tissues, and organs have been established as sustainable alternatives for the controlled production of secondary metabolites. These systems enable isolation of bioactive compounds regardless of environmental conditions, although their efficiency depends on critical variables such as the type of explant, the composition of the culture medium, and the growth regulators used [13,14].

Among the species of interest is *Ludwigia octovalvis* (Onagraceae), commonly known as “clavillo” (peach palm). This plant is mainly distributed in tropical and humid regions. It has been widely used in traditional Mexican medicine, especially in the states of Morelos, Veracruz, and Oaxaca. It is used to treat skin conditions, urinary tract infections, and diabetes [15,16]. In other countries, its use has expanded to include management of hypertension, inflammatory processes, infections, and ulcers [17,18,19]. Phytochemical studies have revealed the presence of flavonoids, phenolic compounds, triterpenes, and steroids, all of which are associated with antimicrobial and wound-healing activity. These compounds participate in processes such as re-epithelialization, fibroblast infiltration, and collagen deposition [20,21,22,23,24,25,26,27,28,29]. Accordingly, this study was oriented toward establishing callus cultures of *L. octovalvis* to produce bioactive compounds, characterize their phytochemical profiles, and evaluate their antimicrobial and healing potential, thus providing evidence of their biotechnological and therapeutic value.

## 2. Results and Discussion

### 2.1. Effect of PGRs on Callus Induction

The morphogenetic potential of leaf and nodal segments was evaluated by exposing them to 49 treatments with plant growth regulators (PGRs) designed from factorial combinations of the cytokinins BAP and KIN and the auxins 2,4-D and NAA. This factorial experimental design enabled systematic evaluation of not only the individual effects of each regulator but also potential synergistic or antagonistic interactions between auxins and cytokinins, providing a comprehensive view of their influence on in vitro morphogenetic induction.

Furthermore, this approach facilitated direct comparison of differential responses among explant types, enabling identification of specific patterns of cell competence and hormonal sensitivity. Thus, it was possible to determine the most efficient combinations for inducing cell dedifferentiation and proliferation and establish the importance of hormonal balance in tissue reprogramming and triggering specific morphogenetic pathways.

The results revealed a marked difference in cell competence across explant types. While nodes showed significant recalcitrance with no or minimal induction responses, only the T18 and T36 treatments with 2.22/2.32 µM BAP/KIN + 5.37 µM NAA promoted callus formation by 25%, indicating that the use of NAA with different auxins but at the same concentration promotes callus induction in node explants; however, this response was minimal compared to that in leaf explants.

Leaf explants exhibited outstanding callus-forming capacity under specific conditions. Analysis of the PGRs individually showed that cytokinins alone induced a marginal response in leaves but no responses in nodes, whereas auxins alone did not promote cell dedifferentiation.

In contrast, the synergism between auxins and cytokinins was crucial for callus induction in most leaf treatments. The data suggest that leaf tissue has a higher density of meristematic cells that interact effectively with PGRs.

According to the statistical evidence (Table 1), the BAP/2,4-D combination proved to be the most efficient for induction. Treatments T22 (0.44/0.45 µM), T24 (0.44/4.52 µM), T26 (2.22/2.26 µM), and T28 (4.44/0.45 µM) achieved induction rates exceeding 87.5%, standing out for generating calluses with optimal friability characteristics, a desirable condition for the subsequent establishment of cell suspensions. In contrast, the combinations with KIN/2,4-D (treatments T40-T45) showed significantly lower efficiency; treatments T40, T46, T47, and T48 showed a 0% induction rate, while treatments T41, T42, T43, T44, and T45 exhibited induction rates ranging from 17.5% to 37.5%. Furthermore, these calluses exhibited suboptimal morphologies characterized by a compact appearance and signs of tissue oxidation.

In conclusion, leaf segments are the ideal explants for callus formation in *L. octovalvis*, with the BAP/2,4-D interaction being the most robust hormonal system with respect to maximizing both the induction rate and biotechnological quality of the callus formed. Likewise, this hormonal combination has also promoted callogenesis in leaf explants of *Lopezia racemosa* Cav. (Onagraceae), using 2,4-D/BAP [30]. On the other hand, in *Oenothera biennis* L. and *Epilobium parviflorum* Schreb. (Onagraceae), optimal callus formation from leaf segments was achieved using the KIN/2,4-D combination [31,32].

Following the evaluation of the induction phase, the cell lines with the highest morphogenic potential, corresponding to treatments T22, T24, T26, T28 (BAP/2,4-D), and T21 (BAP/NAA), were selected for establishment in maintenance stages through periodic subcultures. However, a gradual loss of viability was observed in most lines during the stabilization process, with signs of senescence and cell death.

**Figure 1:**
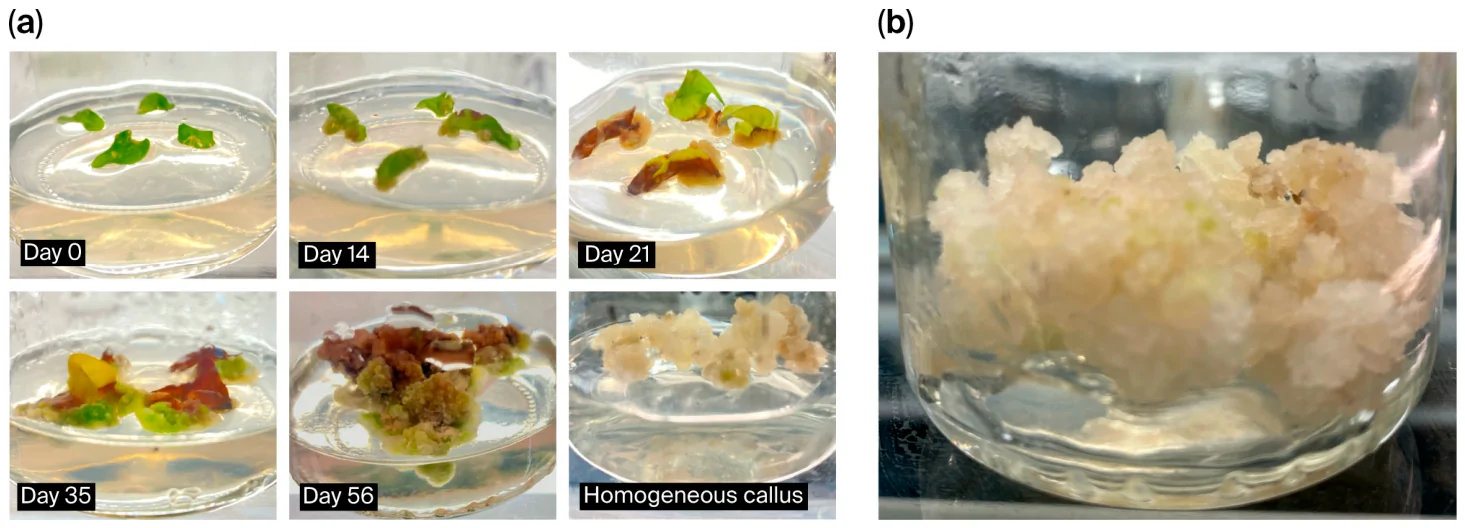
Establishment of callus culture from leaf explants treated with 4.44 µM BAP + 0.45 µM 2,4-D. (**a**) Sequential stages of callus induction and development recorded at 0, 14, 21, 35, and 56 days after culture initiation. The image labeled “Homogeneous callus” corresponds to callus cultures after six successive subcultures performed at 21-day intervals. (**b**) Friable homogeneous callus obtained after the sixth subculture.

Only treatment T28 (4.44 µM BAP + 0.45 µM 2,4-D) led to sustained metabolic resilience and adaptability to subculture conditions. As observed in chronological monitoring (Figure 1), the transition from leaf explant to cell mass formation was consolidated from the fifth week onward, achieving complete biotechnological stabilization after six months of continuous culturing.

Calluses derived from treatment T28 exhibited remarkable phenotypic homogeneity, characterized by a pale-beige coloration and highly friable texture, attributes indicative of active cell division and the absence of significant phenolic oxidation. This cell line reached kinetic equilibrium, requiring 21-day subculture intervals to prevent nutrient limitation and preserve its proliferative potential in advanced subcultures (Subculture No. 6).

The efficiency observed in treatment T28 (4.44 µM BAP and 0.45 µM 2,4-D) for the consolidation of friable calluses in *L. octovalvis* is closely correlated with that reported for explants of *Vitis vinifera* L., since with this same hormonal interaction it is optimal to obtain viable cell biomass [33]. However, the variability in cell competence is evident when comparing these results with those for species such as *Eleusine coracana* (L.) Gaertn., wherein the application of the same ratio of PGRs elicited no callogenic response [34], highlighting the determining role of genotype in cell reprogramming.

Within the Onagraceae family, induction protocols reveal significant heterogeneity in hormonal requirements. While systems based on equimolar concentrations of auxins (9.05 μM of 2,4-D and 10.74 μM of NAA) have been standardized for *Oenothera elata* ssp. *hookeri* and *Oenothera picensis* Phil. [35], other species in the family show a greater affinity for cytokinin–auxin combinations. For example, the use of 4.65 μM of KIN in synergy with low doses of 2,4-D (1.13 μM) has been documented in *Oenothera biennis* L. [31].

The comparison with *Lopezia racemosa* Cav. is particularly relevant, as callus formation was optimized with 5.37 μM 2,4-D and 0.44 μM BAP [30], a ratio that is inversely related to the ideal ratio selected in this study for *L. octovalvis*. These findings corroborate the view that, although there are response patterns within Onagraceae, the determination of PGR concentration and type remains species-specific, and the fine-tuning of the BAP/2,4-D ratio reported here represents a critical advancement for the biotechnological domestication of *L. octovalvis*.

### 2.2. Growth Kinetics of Callus Culture

**Figure 2:**
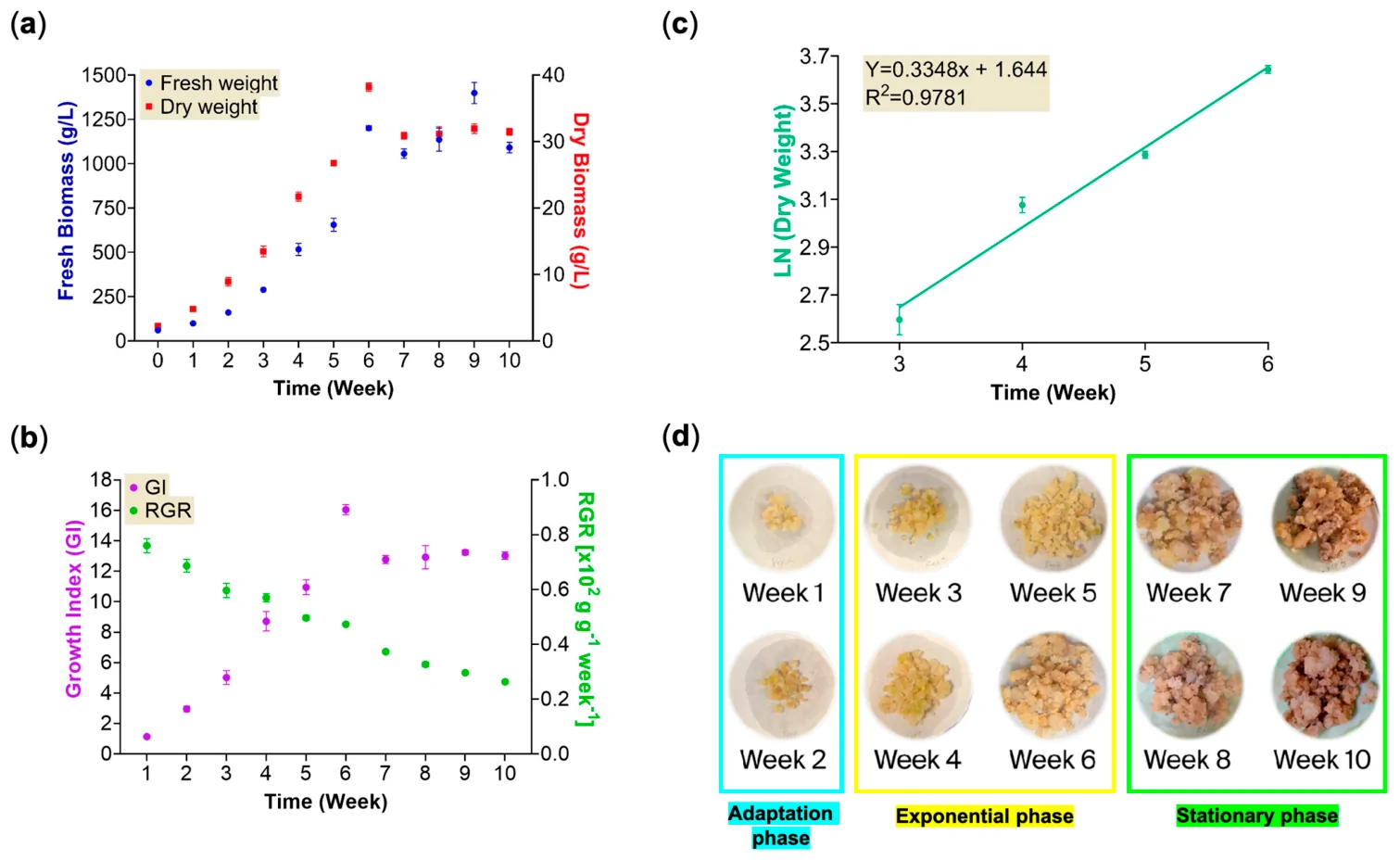
Characteristics of callus growth. (**a**) Growth kinetics based on fresh and dry biomass accumulation over time. (**b**) Growth index (GI) and relative growth rate (RGR) during the culture period. (**c**) Exponential growth curve with linear regression analysis. (**d**) Morphological changes in callus during the culture period, showing the adaptation phase (weeks 1–2), exponential phase (weeks 3–6), and stationary phase (weeks 7–10). Data are presented as mean ± standard error (SE); *n* = 3.

The growth kinetics of T28 calli obtained in a medium supplemented with 4.44 μM BAP and 0.45 μM 2,4-D were examined by measuring increases in fresh and dry biomass as well as the growth index (GI) and relative growth rate (RGR). In addition, photographic monitoring was conducted at each point in the growth kinetics (Figure 2).

The growth curve shows an adaptation phase from weeks 0 to 2, followed by exponential growth from weeks 3 to 6. Maximum dry biomass was observed at week 6 (38.21 ± 0.62 g/L), along with the highest growth index (16.04 ± 0.33). Biomass continued to accumulate, reaching peak fresh biomass at week 9 (1399.03 ± 104.11 g/L) (Figure 2a). After week 9, biomass levels stabilized with growth index ranging from 12.77 to 13.24, marking the stationary phase. These findings indicate that maximum dry matter accumulation occurred before peak fresh biomass production. During this stage, the RGR decreased progressively from 0.76 to 0.26 × 10^2^ g g^−1^ week^−1^, which likely reflects changes associated with callus developmental stages and prolonged culture duration (Figure 2b).

Based on the exponential phase, the maximum specific growth rate (µ_max_) was calculated to be 0.33 week^−1^ (0.047 d^−1^), corresponding to a doubling time of 2.08 weeks (14.54 d) (Figure 2c). These results indicate a moderate capacity for proliferation of *L. octovalvis* calli under the evaluated conditions. In comparison with other in vitro culture systems, doubling times of 9.6 days, 4.7 days, and 5.69 days have been reported in *Taxus baccata* L. callus cultures [36], *Centella asiatica* (L.) Urb. cell suspensions [37], *Panax ginseng* C.A. Mey. adventitious root cultures [38], respectively, suggesting that growth kinetics depend on both the plant species and the type of culture used.

The morphology of the callus also exhibited changes associated with the different growth phases. During the exponential phase, the callus remained a uniform beige color; however, by the sixth week, it had darkened slightly, coinciding with a moderate decrease in GI, indicating the onset of senescence. Finally, during the stationary phase, the callus underwent progressive oxidation, acquiring a brown hue possibly related to cellular aging and the accumulation of phenolic compounds (Figure 2d).

### 2.3. Chemical Analysis of Callus Biomass

The phytochemical profiles of the extracts from the T28 cell line of *L. octovalvis*, analyzed quantitatively via gas chromatography–mass spectrometry (GC-MS), enabled the unequivocal separation and identification of 15 primary and specialized metabolites (Table 2). The distribution and yield of these compounds showed a direct dependence on the polarity and dielectric constant of the solvents used in fractionation, ranging from the nonpolar environment of *n*-hexane and the intermediate polarity of ethyl acetate to the highly hydrophilic system of methanol. Within this chemotaxonomic landscape, only *n*-hexadecanoic acid, oleic acid, and the phytosterol γ-sitosterol exhibited metabolic ubiquity, which was consistently detected across all three extraction phases, although their relative abundances varied significantly depending on their affinity for each solvent matrix. Detailed analysis of each fraction revealed that the lipophilic extract obtained with *n*-hexane was characterized by a preferential accumulation of structural lipids, fatty acids, and long-chain steroidal compounds. In this low-polarity phase, γ-sitosterol emerged as the predominant metabolite, with a relative abundance of 17.42%, followed by 7-dehydrodiosgenin, a steroidal saponin, which reached 9.89%, suggesting efficient activation of the mevalonate pathway in callogenic cells. In smaller proportions, oleic acid (2.85%), 1-heptatriacontanol (1.20%), *n*-hexadecanoic acid (1.11%), isopropyl palmitate (0.84%), and palmitoleic acid (0.74%) were identified.

The ethyl acetate extract, representative of intermediate polarity, served as a transition matrix that solubilized residual steroidal compounds, fatty acids, and carbohydrates. In this fraction, γ-sitosterol and 7-dehydrodiosgenin were found in high abundances (12.80% and 11.96%, respectively). The highest concentration of *n*-hexadecanoic acid (11.74%) was also observed in this phase. The chemical diversity of the extract was further enhanced by the presence of stigmasterol (6.76%), D-mannose (4.32%), cellobiose (3.08%), and oleic acid (4.87%). Trace amounts of biologically relevant compounds were also detected, including desulfosinigrin (0.72%), β-sitosterol (0.59%), and methyl 6-oxoheptanoate (0.28%).

In contrast, the methanolic extract exhibited a markedly hydrophilic profile characterized by the absence of most long-chain lipophilic compounds and the predominance of oxygenated heterocycles derived from carbohydrate metabolism. The major compound in this phase, and among all metabolites detected, was 5-hydroxymethylfurfural, with a relative abundance of 39.89%, followed by 2,3-dihydro-3,5-dihydroxy-6-methyl-4H-pyran-4-one (15.50%) and D-mannose (14.86%). In smaller proportions, methanol also extracted *n*-hexadecanoic acid (5.56%), γ-sitosterol (2.27%), oleic acid (2.09%), and cellobiose (0.83%). This partial overlap in chromatographic profiles supports the callus biofactory’s potential as a viable and sustainable alternative for producing secondary metabolites of pharmacological interest, thereby reducing dependence on wild-population collections.

Analytical characterization of the in vitro biomass revealed a remarkable metabolic congruence with native *L. octovalvis* specimens reported in the scientific literature. The presence of compounds such as oleic acid, stigmasterol, palmitoleic acid, *n*-hexadecanoic acid, γ-sitosterol, and β-sitosterol confirms that the dedifferentiated somatic cells from treatment T28 maintain the main biosynthetic pathways of the wild-type organism [28,29,39,40]. However, the culture conditions also induced marked metabolic plasticity, promoting alternative pathways for the synthesis of compounds characteristic of the Onagraceae family, such as desulfosinigrin, 7-dehydrodiosgenin, 5-hydroxymethylfurfural, and the pyran derivative DDMP [41,42,43,44,45,46,47,48,49,50,51,52,53,54,55]. This partial match, both qualitatively and quantitatively, supports the potential of this cell line as a controlled, homogeneous, and sustainable biofactory for producing phytochemicals of interest, thereby reducing the need to exploit wild populations.

**Figure 3:**
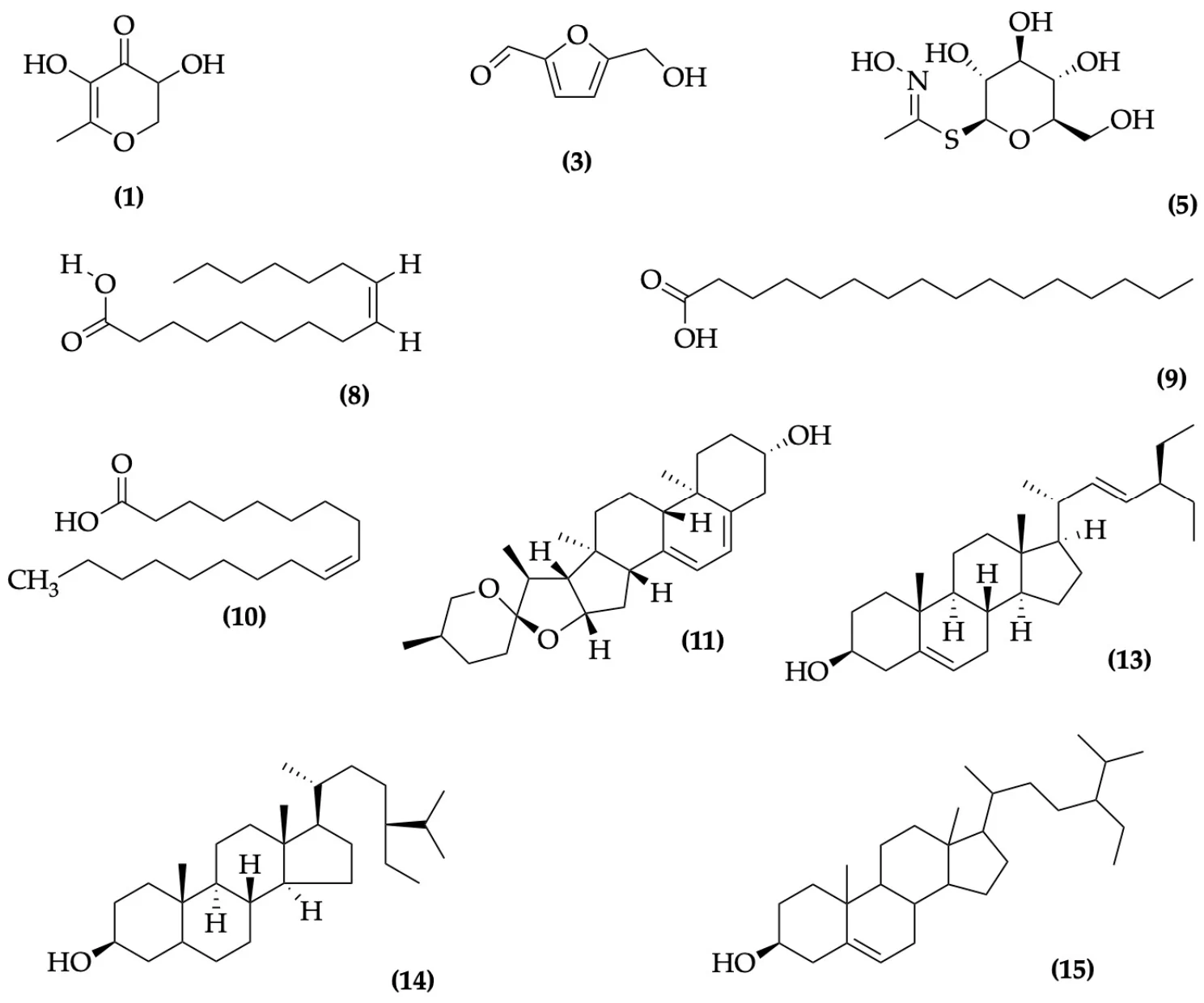
Chemical structures of compounds relevant for their reported therapeutic potential, identified by GC-MS in hexane, ethyl acetate, and methanol extracts from callus cultures subjected to the T28 treatment (Table 2). Structures were generated using ChemDraw.

Finally, the pharmacological attractiveness of this biomass lies in the presence of several major metabolites identified, including furan and pyran derivatives, desulfosinigrin, unsaturated fatty acids, and phytosterols, which are widely recognized for their biological activity (Figure 3). Various studies have demonstrated that these compounds possess antioxidant, antimicrobial, anti-inflammatory, anticancer, cytotoxic, and antifungal properties, frequently mediated by synergistic mechanisms. In this context, the callus culture system optimized with T28 treatment emerges as a robust biotechnological platform for large-scale production of bioactive compounds for medical applications and the development of phytopharmaceuticals. The chemical structures shown in Figure 3 were generated using ChemDraw (26.0.0.6599) based on the compounds identified by GC-MS.

### 2.4. PASS Predictions Activities

Predictive evaluation using the PASS (Prediction of Activity Spectra for Substances, Version 2.0) software for metabolites identified in the *L. octovalvis* T28 cell line revealed a pleiotropic pharmacological profile, with notable convergence toward antimicrobial and wound-healing properties. The results suggest that this chemoprofile acts synergistically on multiple molecular targets that regulate redox homeostasis, the immunomodulatory response, and the kinetics of tissue regeneration.

Regarding chemotherapeutic potential (Table 3), the predictions indicate a predominance of antiviral activity, complemented by antibacterial and antifungal effects. At the mechanistic level, several compounds were linked to the disruption of critical biosynthetic processes, such as peptidoglycan polymerization and the fidelity of nucleic acid replication/transcription, as well as the inhibition of fungal cell wall structural enzymes. Furthermore, the anticipated inhibitory effect on general pumps suggests interference with membrane-associated transport systems, including microbial efflux pumps, which are essential for cellular homeostasis and often contribute to antimicrobial resistance. These metabolic targets are fundamental for pathogen viability and typically associated with bactericidal mechanisms of action [56,57].

However, it is imperative to clarify that metabolites targeting enzymes involved in intermediary metabolism or virulence factors could exert bacteriostatic or anti-virulence effects. Therefore, although in silico analysis provides a robust basis for biocompound prospecting, this interpretation is considered preliminary. Final therapeutic efficacy depends on key experimental variables, including the minimum inhibitory concentration (MIC), the susceptibility of the evaluated serotype, and bioavailability within the cellular microenvironment, factors that require rigorous in vitro and in vivo validation [58].

Predictive analysis revealed that the metabolites identified converge on critical biological axes spanning the inflammatory, proliferative, and tissue-remodeling phases (Table 4). Within the proliferative dynamic, compounds 8, 9, and 10 stand out for their ability to stimulate angiogenesis and act as agonists of the fibroblast growth factor (FGF) receptor; these activities are crucial for neovascularization and fibroplasia, processes orchestrated primarily by the VEGF and FGF signaling pathways [59,60,61]. This action is complemented by an immunomodulatory and homeostatic response, in which the efficient transition through the inflammatory phase is supported by the stimulation of macrophages and leukopoiesis, mainly attributed to compound 10, along with the regulation of erythropoiesis mediated by compounds 10 and 13, ensuring the removal of cellular debris and optimal oxygen delivery to the injured tissue.

In parallel, cellular robustness against the cytotoxic microenvironment of the wound is reinforced by the cytoprotective and mucomembrane properties of metabolites 1, 8, 13, 14, and 15. In particular, the induction of HMOX1 expression by compounds 10 and 13, along with the maintenance of membrane integrity mediated by compounds 1, 3, and 8, suggests a robust defense against oxidative stress and lipid peroxidation. Finally, we report a critical finding, namely, the modulation of the extracellular matrix, in which compounds 8 and 9 modulate collagen maturation by acting as inhibitors of procollagen endopeptidases (C and N) and procollagen-proline dioxygenase. This enzymatic regulation is essential for controlling fiber polymerization and preventing aberrant fibrotic remodeling, thus facilitating functional wound closure [62]. Taken together, the specific classification of compounds 14 and 15 as healing agents, coupled with the vasoprotective support of metabolites 1 and 9, underscores the therapeutic potential of *L. octovalvis* callus in regenerative medicine.

Taken together, these findings support the hypothesis that secondary metabolites derived from the T28 callus of *L. octovalvis* exert multifactorial biological activity by modulating redox homeostasis, immune responses, and molecular signaling within tissue regeneration pathways. This interaction, combined with their potential for microbial inhibition, positions these extracts as promising candidates for developing complex phytotherapeutic agents.

It is imperative to clarify that the predictions obtained with the PASS software (Way2Drug) are probabilistic and based on structure–activity relationships and similarity to pre-existing bioactive chemotypes [63]. Consequently, while these bioinformatics tools offer valuable mechanistic guidance, the results they yield do not constitute definitive confirmation of biological efficacy or in vivo pharmacodynamics. In this context, systematic experimental validation through functional bioassays and cell models is essential to corroborate the suggested therapeutic potential and elucidate the underlying mechanisms of action of these compounds.

### 2.5. Antibacterial Activity

The antibacterial activity of callus extracts obtained under T28 treatment was evaluated against *S. aureus*, methicillin-resistant *S. aureus* (MRSA), and *P. aeruginosa* (Table 5). Among the tested extracts, the methanolic extract (T28-MeOH) exhibited the highest antibacterial activity, with MIC values of 31.25 µg/mL against *S. aureus* and 62.5 µg/mL against MRSA. In contrast, the ethyl acetate extract (T28-EtOAc) showed moderate activity, with MIC values of 500 and 250 µg/mL, respectively, while the hexane extract (T28-Hex) was inactive (MIC > 1000 µg/mL). None of the extracts showed inhibitory activity against *P. aeruginosa*. Gentamicin exhibited strong antibacterial activity against all tested strains.

The higher activity observed in the methanolic extract may be associated with the presence of polar bioactive and semi-polar compounds identified via GC–MS, including 5-hydroxymethylfurfural and 2,3-dihydro-3,5-dihydroxy-6-methyl-4H-pyran-4-one, which have been reported to exhibit antimicrobial properties [44,64]. Additionally, fatty acids such as oleic acid and *n*-hexadecanoic acid, together with phytosterols including stigmasterol and sitosterols, may contribute to the overall antibacterial effect through possible membrane-disrupting and synergistic mechanisms [53,65,66].

The MIC of 31.25 µg/mL exhibited by T28-MeOH against *S. aureus* falls within the range generally regarded as strong antibacterial activity for crude plant extracts (commonly defined as an MIC < 100 µg/mL), and the activity against MRSA (62.5 µg/mL) is particularly relevant given the clinical burden imposed by methicillin resistance [67,68,69]. These values are consistent with and extend previous reports of antibacterial activity of wild *L. octovalvis*, both in plant extracts and in products derived from this species, against *S. aureus* and other relevant pathogens indicating that the T28 callus reproduces the antibacterial trait of the source plant under controlled in vitro conditions.

The lack of antibacterial activity against *P. aeruginosa* and *E. coli* may be attributed to the intrinsic structural and physiological characteristics of Gram-negative bacteria. The outer membrane, enriched in lipopolysaccharides, acts as a permeability barrier that limits the penetration of many phytochemicals, particularly hydrophobic constituents. Consequently, Gram-negative species typically exhibit lower susceptibility to plant extracts rich in fatty acids than Gram-positive organisms [70,71]. Furthermore, efflux pump systems can actively reduce intracellular accumulation of bioactive compounds, decreasing their effective concentration at target sites [72]. In contrast, Gram-positive bacteria lack this outer membrane, facilitating the interaction of lipophilic compounds with the cytoplasmic membrane, often resulting in higher susceptibility to plant-derived extract [73].

Overall, these results suggest that callus cultures treated with T28 constitute a promising source of antibacterial metabolites, particularly against Gram-positive bacteria.

### 2.6. Wound Evaluation and Closure Percentage

**Figure 4:**
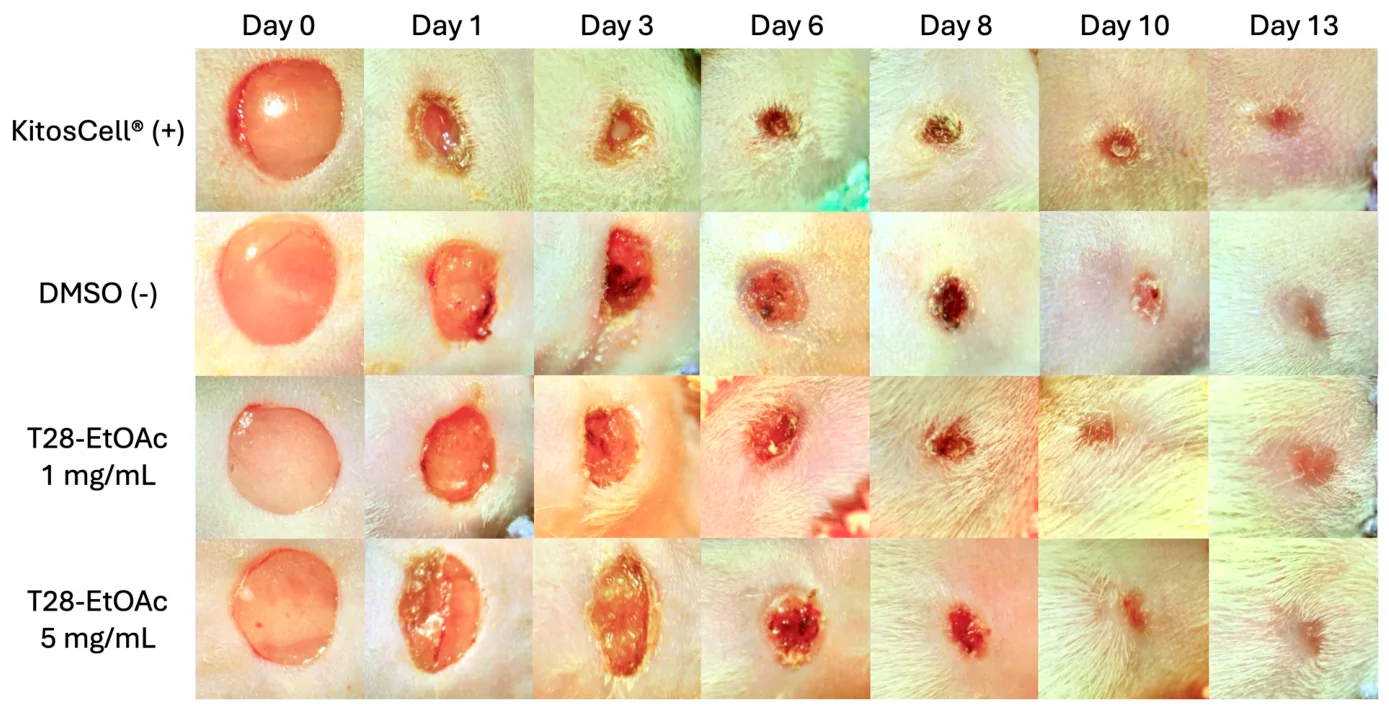
Photographic progression of wound healing over a 13-day period. Positive control group (KitosCell^®^); negative control group without treatment (1% DMSO); T28-EtOAc—ethyl acetate extract of T28 callus (*n* = 5 in each group).

Macroscopic evaluation of the healing process in full-thickness excision wounds was performed sequentially over a critical 13-day period, enabling precise characterization of tissue closure dynamics under different experimental conditions (Figure 4). The variations observed in the progressive reduction in wound area reflected specific pharmacological modulation across the phases of inflammation, proliferation (including fibroplasia and re-epithelialization), and extracellular matrix remodeling, clearly differentiating the profiles of the control groups from those of the group treated with the ethyl acetate extract of T28 calluses (T28-EtOAc).

In the positive control group, which was treated with KitosCell^®^ (pirfenidone-based formulation), accelerated and highly efficient healing kinetics were evident. In the first few days, rapid contraction of the wound area was observed, correlating with an accelerated transition from the hemostatic phase to the proliferative phase. Early crust formation between days one and three ensured protection of the wound bed and the early initiation of re-epithelialization. By day six, the reduction in lesion diameter was statistically significant, progressing to near-complete closure by day thirteen, characterized by minimal residual inflammation and organized tissue architecture. This behavior validates the experimental model and confirms that the modulation of profibrotic cytokines, particularly TGF-β, is a key mechanism in the accelerated resolution of the tissue defect [74].

In contrast, the negative control group treated with 1% DMSO showed a marked delay in the physiological phases of healing. During the initial days, the wound bed exhibited persistent blood clots, indicating delayed resolution of the hemostatic and inflammatory phases and poor fibroblast recruitment. The degree of tissue contraction was significantly less relative to the positive control, and crust formation was delayed, occurring only around day six. However, the negative control group treated with 1% DMSO exhibited a marked delay in the physiological phases of healing. During the initial days, the wound bed showed persistent blood clots and reduced tissue contraction compared with the treated groups. Crust formation was also delayed, occurring around day six. Although substantial wound closure was observed by day 13, the DMSO group maintained a residual wound area significantly greater than that of the treated groups, indicating that endogenous repair mechanisms alone were less efficient in promoting tissue restoration during the evaluation period.

Topical treatments with the T28-EtOAc extract induced notable improvements in healing, with a dose-dependent dynamic suggesting differential mechanisms of cell interaction. At a concentration of 1 mg/mL, the extract promoted a progressive, steady reduction in wound area starting on day three, accompanied by adequate scab formation and moderate contraction of the surrounding tissue. Advanced closure was achieved by day ten, suggesting there was effective stimulation of keratinocyte migration and basal fibroblast proliferation. In contrast, the 5 mg/mL concentration exhibited biphasic kinetics: although the initial contraction was slower, by day three, early scab formation was established, triggering an accelerated proliferative phase. Between ten and thirteen days, wounds treated with this concentration achieved almost complete closure, with mature granulation tissue, re-epithelialization, and a homogeneous skin surface, making them comparable to the clinical reference standard.

A mechanistic interpretation of these findings suggests that the T28-EtOAc-mediated acceleration of wound closure can be explained using a multi-objective approach. First, the early transition to crust formation mitigated fluid loss and protected the wound bed from secondary infections, thus contrasting with the persistence of unstable clots in the DMSO group. Second, the significant reduction in wound area from day six onward suggests that secondary metabolites of the extract induce differentiation of fibroblasts into myofibroblasts, cells characterized by α-SMA expression and responsible for the contractile forces that approximate the wound edges [75]. Finally, the 5 mg/mL concentration promoted balanced collagen synthesis and adequate angiogenesis, facilitating the coordinated migration of keratinocytes onto a healthy, vascularized granulation tissue, contrasting with the disorganized matrix observed in the negative control.

Therefore, the macroscopic evaluation and the kinetics of wound closure percentage rigorously demonstrate the regenerative properties of the T28-EtOAc extract. The 5 mg/mL dose stands out for triggering an accelerated healing response with high morphological quality comparable to reference clinical treatments.

Our analysis of the temporal evolution of wound closure reveals a statistically significant (*p* < 0.05, *n* = 5) differential modulation of tissue repair kinetics by the administered treatment (Table 6).

This biological process was clearly structured in three successive physiological phases during the 13 days of evaluation. During Phase I, which spanned days 0 to 3 and correlated with hemostasis and early inflammatory events, a clear bifurcation in the cellular response was observed. The groups treated with the low-concentration ethyl acetate extract (T28-EtOAc, 1 mg/mL) and the positive control (KitosCell^®^) exhibited robust and parallel acceleration from the first 24 h, achieving over 50% wound contraction by day 1 and approximately 64% and 77% by day 3, respectively. Conversely, both the negative control group (1% DMSO) and the high-dose treatment group (T28-EtOAc 5 mg/mL) exhibited a pronounced kinetic delay in this initial stage, remaining below 30% closure at the end of the first phase. This divergence suggests that the 1 mg/L concentration has biphasic or modulatory activity that mitigates the chronic inflammatory phase and stimulates early cell migration of keratinocytes and fibroblasts, emulating the efficacy of the reference drug, while the 5 mg/mL dose can exert a receptor saturation effect or mild, may suggest receptor saturation or a transient’s inhibitory effect. Subsequently, during Phase II, or the proliferative phase (days 4 to 8), the acceleration rates of the treatment dynamics reversed. The group treated with T28-EtOAc 5 mg/mL showed the most abrupt increase in the slope of the curve between days 3 and 6, going from an initial lag to reaching a remarkable 74% closure by day 6 and then converging with the positive control by day 8 at approximately 91%. Meanwhile, the T28-EtOAc extract at 1 mg/mL maintained its steady upward progression, achieving almost complete closure (~98%) by day 8, a value numerically higher than that of all other groups evaluated, including the positive control. This differentiated behavior indicates that while the 5 mg/mL concentration delays early events, it acts selectively and potently as a promoter of fibroplasia, extracellular matrix synthesis, and re-epithelialization once the proliferative phase is established.

Finally, Phase III (days 9–13), corresponding to tissue remodeling and maturation, revealed clear differences among treatments. Both concentrations of T28-EtOAc and the positive control achieved almost complete macroscopic wound closure by day 13, whereas the DMSO group retained a residual wound area of 0.32%. These findings indicate that the extract accelerated not only the early phases of repair but also promoted a more effective completion of the healing process than spontaneous recovery alone. This differential response compared to the positive control may be associated with the antifibrotic activity of pirfenidone, mediated by the modulation of profibrotic cytokines and key growth factors, including transforming growth factor beta (TGF- β), suggesting a potentially more sustained regulation of the extracellular-matrix-remodeling phase [76].

Skin wound healing is a highly coordinated, dynamic, and kinetically overlapping physiological process involving an intricate cascade of cellular, biochemical, and molecular events aimed at restoring tissue barrier integrity [77]. Classically, this phenomenon is divided into four interconnected temporal microenvironments: hemostasis, inflammation, proliferation, and tissue remodeling. The cascade begins with hemostasis, whose immediate goal is to mitigate vascular compromise by activating the coagulation pathway, promoting platelet aggregation, and initiating the assembly of a provisional fibrin matrix. In this initial stage, degranulated platelets act as epicenters of paracrine signaling, releasing chemokines and critical growth factors—such as TGF-β and platelet-derived growth factor (PDGF)—that orchestrate the chemotactic recruitment of cell lineages to the wound bed [78,79].

**Figure 5:**
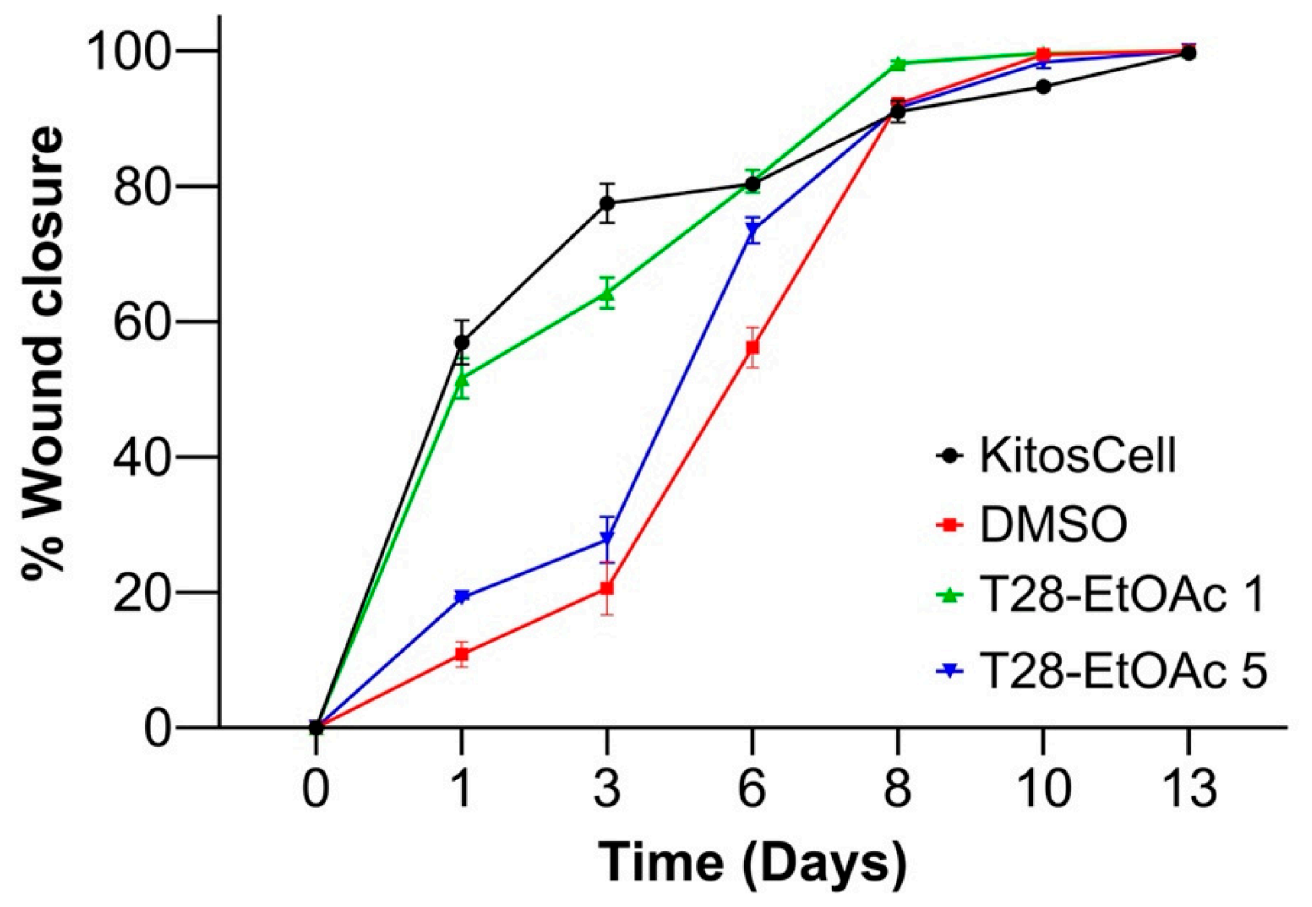
Temporal evolution of the wound area. Positive control group (KitosCell^®^); negative control group without treatment (1% DMSO); T28-EtOAc—ethyl acetate extract of T28 callus. Data are expressed as the mean ± standard error (*n* = 5).

The transition to the inflammatory phase (days 0–3, denoted as Phase I in Figure 5) is mediated by the sequential infiltration of polymorphonuclear leukocytes (neutrophils) and circulating monocytes, which differentiate locally into macrophages. These cells clear microbial and cellular debris through phagocytosis and an oxidative burst while secreting proinflammatory cytokines and transcription factors that modulate the adaptive response of the damaged niche [61,80].

Subsequently, the proliferative phase or Phase II (days 4–8) activates angiogenesis, fibroplasia, and re-epithelialization. During this window, local fibroblasts migrate and proliferate in response to the growth factor gradient, actively synthesizing essential components of the extracellular matrix (ECM), such as primarily type III collagen, while keratinocytes at the peri-lesional margins undergo an epithelial–mesenchymal transition, proliferating and migrating horizontally to promote physical wound closure [81,82]. Finally, the remodeling phase, or Phase III (days 9–13), constitutes the tissue maturation stage, characterized by apoptosis of redundant cells and the replacement of type III collagen with type I collagen fibers organized into parallel bundles with high tensile strength. This architectural scaffolding process depends closely on a strict homeostatic balance between the catalytic activity of matrix metalloproteinases (MMPs) and their respective tissue inhibitors of metalloproteinases (TIMPs) [77,83,84].

In this study, the pharmacological evaluation of the ethyl acetate extract of T28 callus (T28-EtOAc) revealed a differential, concentration-dependent modulation of the kinetic transitions in the healing process. Administration of T28-EtOAc at a low dose of 1 mg/mL robustly accelerated wound closure during the early inflammatory phase (Phase I), achieving significantly greater lesion contraction (*p* < 0.05) than the negative control and matching the kinetics of the commercial positive control (KitosCell^®^). This phenotypic response suggests that the 1 mg/mL concentration optimizes the resolution of the acute inflammatory cascade, preventing the tissue from progressing to chronic restrictive inflammation and promoting an early transition to cell recruitment. In marked contrast, the 5 mg/L dosage induced an initial kinetic lag during Phase I but led to an exponential increase in the contraction rate (maximum slope of the curve) during the proliferative phase (Phase II, days 4–8), efficiently converging with the groups with the highest closure rates by day 8. This biphasic behavior indicates that the extract’s phytoconstituents act through distinct mechanisms depending on their concentration thresholds: while lower doses optimally attenuate early inflammatory events, higher concentrations trigger a secondary mitogenic and trophic stimulus that selectively accelerates fibroplasia and neovascularization in the granulation tissue.

This multi-phase regulatory phenomenon is closely correlated with the body of scientific evidence that positions plant-derived secondary metabolites as allosteric modulators and efficient ligands of skin repair signaling pathways. Terpenoids, saponins, phenolic compounds, and phytosterols have been widely reported to exert pleiotropic effects that mitigate oxidative stress, inhibit pro-inflammatory pathways, and transcriptionally stimulate collagen synthesis and tissue remodeling [85,86,87,88]. Collectively, these metabolites can modulate multiple cellular and molecular mechanisms involved in wound repair and healing. Collectively, this network of secondary metabolites can interconnect multiple molecular targets within the wound microenvironment.

From a chemotaxonomic perspective, the phytochemical profile of T28-EtOAc, determined via GC-MS, provides a robust molecular basis for the therapeutic effects observed. The presence of long-chain fatty acids such as *n*-hexadecanoic acid (palmitic acid) and oleic acid, along with a prominent sterol fraction composed of stigmasterol, γ-sitosterol, and β-sitosterol, supports the biological activity of the extract. At the molecular level, *n*-hexadecanoic acid mitigates the acute inflammatory phase by competitively inhibiting soluble phospholipase A2 (sPLA2), thereby blocking the release of arachidonic acid from cell membrane phospholipids and subsequently suppressing the catalytic activity of cyclooxygenase-1 (COX-1) and cyclooxygenase-2 (COX-2) enzymes [54,89]. Synergistically, oleic acid acts as a potent modulator of the wound microenvironment in vivo; it directly stimulates re-epithelialization by accelerating keratinocyte migration and regulates the expression of local inflammatory mediators, acting in parallel as a complementary repressor of the phospholipase A2 cascade [90,91].

Furthermore, the phytosterol fraction identified in the T28-EtOAc extract provides crucial mechanistic support for resolving phases II and III of wound healing. Stigmasterol attenuates tissue damage mediated by chronic inflammation by suppressing key pro-inflammatory mediators and inhibiting the transcriptional expression of inducible nitric oxide synthase (iNOS) and COX-2 [92]. β-sitosterol, for its part, exerts robust control over the NLRP3 inflammasome and the nuclear factor kappa light chain enhancer (NF-κB) signaling pathway, resulting in a drastic downregulation of crucial cytokines such as tumor necrosis factor alpha (TNF-α) and pro-inflammatory interleukins (IL-1β, IL-6, and IL-8) [50]. Beyond its anti-inflammatory role, β-sitosterol is directly involved in Phase II by promoting angiogenesis, inducing collagen synthesis, and favoring the polarization of resident macrophages toward the M2 (anti-inflammatory/repair) phenotype, a critical neuroendocrine step for granulation tissue maturation [93]. Finally, γ-sitosterol complements this bioactive spectrum by demonstrating potent systemic and local anti-inflammatory activity in vivo, as validated in molecular models of edema and leukocyte recruitment [49].

In conclusion, the remarkable regenerative capacity of the T28-EtOAc extract cannot be attributed to a single chemical entity but rather to a multi-target, synergistic pharmacodynamic effect. In this system, fatty acids modulate the initial inflammatory threshold (Phase I), while phytosterols act in concert to enhance cell proliferation, ECM deposition, and angiogenesis (Phase II), converging in an integrated manner toward accelerated, orderly, and functional healing of skin wounds.

## 3. Materials and Methods

### 3.1. Plant Material

A *Ludwigia octovalvis* plant was collected on 7 September 2022 in Lomas de Zomplantle, Morelos, Mexico. Taxonomic identification was performed by Biol. Gabriel Flores Franco, and the seeds were deposited in the Herbarium of the Autonomous University of the State of Morelos (HUMO), Mexico, under accession number 39,816.

### 3.2. Callus Induction and Maintenance

Seed collection from *L. octovalvis* was based on external morphology, with a preference for intact seeds with a uniform dark brown color as an indicator of apparent maturity. The seeds were surface-sterilized under aseptic conditions under a laminar-flow hood. First, the seeds were washed with sterile distilled water containing 0.5% *v*/*v* Axión^®^ surfactant under constant agitation for 15 min and then treated with 1% (*v*/*v*) sodium hypochlorite (Cloralex^®^) for 20 min. Subsequently, the seeds were rinsed three times with sterile distilled water for 5 min each wash.

Finally, the seeds were inoculated into culture tubes (with six seeds per tube, 25 × 150 mm) containing sterile Murashige and Skoog (MS) full strength MS supplemented with 3% sucrose (15 mL per tube), adjusted to pH 5.7, and solidified with 0.3% Phytagel^®^, with no PGRs [94]. The medium was autoclaved at 121 °C and 15 psi for 15 min prior to use. The culture tubes were incubated at 25 ± 2 °C under a 16 h photoperiod with white, fluorescent light (50 μmol m^−2^ s^−1^). This process was repeated three times.

After germination (48 h), the seedlings were maintained for seven days in the same culture medium and then transferred to 6.5 cm tall flasks (25 mL of medium). Following three weeks of in vitro growth, we subcultured the explants in 12.5–15 cm long flasks (100 mL of medium), using the same culture medium described previously at all stages.

Thirty-day-old in vitro-grown plantlets were used as the source of leaf and nodal explants. These explants were transferred to culture dishes (6 × 6.5 cm) containing 25 mL of MS medium with 3% sucrose and 3 g/L of phytagel. This medium was also supplemented with PGRs, either individually or in combination. The PGRs used were: 6-benzylaminopurine (BAP; ≥98%; Sigma-Aldrich, Saint Louis, MO, USA), kinetin (KIN; ≥96%; Sigma-Aldrich, Saint Louis, MO, USA), 2,4-dichlorophenoxyacetic acid (2,4-D; 95%; Sigma-Aldrich, Saint Louis, MO, USA) and naphthaleneacetic acid (NAA; 97%; Sigma-Aldrich, Saint Louis, MO, USA). The PGR concentrations were added to the medium according to a treatment matrix (Table 1). A control without PGR (T0) was included; for BAP, concentrations of 0.44, 2.22, and 4.44 μM were evaluated; for KIN, concentrations of 0.46, 2.32, and 4.65 μM; 2,4-D at 0.45, 2.26, and 4.52 μM; and NAA at 0.54, 2.69, and 5.37 μM were evaluated.

The experimental design comprised 49 treatments, each with five replicates per explant type and four explants per flask, resulting in a total of 40 explants per treatment. The percentage of callus induction was evaluated after 56 days of culture. It was calculated as follows:

% Callus induction = (N° of explants with induced callus/Total n° of cultured explants) × 100(1)

Once a stable callus line was obtained after six months of subculturing at 21-day intervals, callus cultures were thereafter maintained by subculturing every 42 days under the same culture conditions. All subcultures were performed under the same culture conditions: MS medium supplemented with 3% sucrose, 4.44 μM BAP, and 0.45 μM 2,4-D, with the pH adjusted to 5.7 and gelled with 3 g/L Phytagel. Incubation conditions were 25 ± 2 °C under a 16 h photoperiod with cool white fluorescent light (50 μmol m^−2^ s^−1^).

### 3.3. Growth Kinetics

Growth kinetics were determined in batch cultures over a 10-week period (weeks 0–10). For this purpose, 0.5–0.6 g of a 23-day-old callus was inoculated into Gerber-type glass flasks containing 25 mL of MS medium supplemented with the corresponding PGR.

Growth was assessed weekly using the biomass from three independent flasks. Fresh and dry biomass were determined gravimetrically, and based on these values, the growth index (GI) was calculated using the following equation [95,96]:

GI = (W_t_ − W_0_)/W_0_ (2)

In addition, the relative growth rate (RGR) was estimated from dry biomass using the equation below [97,98]:

RGR = (In W_t_ − In W_0_)/(t − t_0_) (3)

where W_t_ is the dry weight at time t, W_0_ is the initial dry weight, and t − t_0_ represents the time interval in days.

GI and RGR were calculated based on dry biomass accumulation to minimize the influence of water content variation in callus tissues.

The maximum specific growth rate (µmax) was obtained via linear regression of the exponential phase of dry biomass growth. From this parameter, the doubling time (t_D_) was calculated using the following equation [97,98,99,100]:

t_D_ = In(2)/μ (4)

### 3.4. Biomass Extraction

The stable callus derived from treatment T28 (42 days old) was used as the source of secondary metabolites. The callus was harvested and dried in an oven at 40 °C. The resulting dry biomass (10.47 g) was ground and subjected to sequential extraction by maceration at room temperature using solvents of increasing polarity (hexane (Hex), ethyl acetate (EtOAc), and methanol (MeOH)) in three successive 72 h stages.

The extracts were filtered through Whatman No. 1 filter paper and concentrated to the point of dryness under reduced pressure using a digital rotary evaporator (model D-402-10; PRENDO, Puebla, Mexico).

### 3.5. GC/MS Analysis

The chemical profile of the T28 extracts (Hex, EtOAc, and MeOH) was analyzed via gas chromatography–mass spectrometry (GC–MS) using an Agilent 6890 system coupled to a 5973N mass selective detector (Agilent Technologies, Santa Clara, CA, USA). Separation was carried out on an HP-5MS capillary column (30 m × 0.25 mm i.d., film thickness of 0.25 µm).

The oven temperature program was initiated at 40 °C for 1 min, followed by a linear increase to 250 °C. The mass spectrometer was operated in electron impact (EI) mode at 70 eV, with an electron multiplier voltage of 1859 V. The oven temperature program started at 40 °C, which was held for 1 min, followed by a linear increase at a rate of 10 °C/min up to 250 °C, with a final holding time of 5 min.

Compound identification was performed by comparing mass spectra with the NIST library (version 1.7a), using a similarity threshold of ≥80% and confirmation based on characteristic fragmentation patterns. Relative quantification was calculated using the peak area normalization method and expressed as percentage abundance (%).

### 3.6. In Silico Prediction of Activity Spectra for Substances (PASS)

The biological activities of the compounds identified in the callus cultures of *L. octovalvis* were predicted using PASS (Prediction of Activity Spectra for Substances) software [101]. The compounds analyzed included 2,3-dihydro-3,5-dihydroxy-6-methyl-4H-pyran-4-one, 5-hydroxymethylfurfural, 7-dehydrodiosgenin, palmitoleic acid, *n*-hexadecanoic acid, oleic acid, stigmasterol, *γ*-sitosterol, *β*-sitosterol, and desulfosinigrin, which were previously identified via GC–MS in hexane, ethyl acetate, and methanolic extracts obtained from callus cultures derived from treatment T28.

PASS is a computational tool used to predict a wide range of pharmacological activities, including antimicrobial and wound-healing properties, based on structure–activity relationship (SAR) analysis. The system is trained on a dataset comprising more than 205,000 compounds associated with over 3750 types of biological activities. For each compound, PASS estimates the probabilities of activity (Pa) and inactivity (Pi). Only activities with Pa values higher than Pi were considered potentially significant; additionally, a threshold of Pa ≥ 0.5 was applied to identify compounds with a moderate-to-high probability of exhibiting antimicrobial and wound-healing activity [101,102,103,104].

### 3.7. Antibacterial Assay

#### 3.7.1. Microorganisms

The antibacterial activity of the T28-derived extracts was evaluated against *Staphylococcus aureus* (ATCC 6538), *Methicillin-Resistant Staphylococcus aureus* (ATCC 43300), *Escherichia coli* (ATCC 8739), and *Pseudomonas aeruginosa* (clinical isolates). The microorganisms were incubated under aerobic conditions at 37 °C.

#### 3.7.2. Broth Dilution Method

Antibacterial susceptibility was evaluated using the broth microdilution method in accordance with the Clinical and Laboratory Standards Institute (CLSI) guideline M100 [105,106]. The T28 extracts (Hex, EtOAc, and MeOH) were tested at concentrations ranging from 31.25 to 1000 μg/mL. Stock solutions were initially prepared in 10% (*v*/*v*) dimethyl sulfoxide (DMSO) (≥99.9%, Sigma-Aldrich, Saint Louis, MO, USA) and subsequently diluted in Mueller–Hinton broth (Difco^TM^, Becton Dickinson, Franklin Lakes, NJ, USA).

Gentamicin (Sigma-Aldrich^®^, St. Louis, MO, USA) was used as a positive control at concentrations of 0.15 to 40 μg/mL, while 10% (*v*/*v*) DMSO served as the negative control. The bacterial inoculum was prepared in 0.85% saline solution and adjusted to the 0.5 McFarland standard. It was then diluted to a final concentration of 5 × 10^5^ CFU/mL.

Each microtiter plate included growth controls (broth plus inoculum) and sterility controls (broth only). For the experimental conditions, 100 μL of bacterial suspension was mixed with 100 μL of each solution. All assays were performed in duplicate, with incubation at 37 °C for 24 h. Absorbance was then measured at 600 nm using a Glomax^TM^ microplate reader (Promega, Madison, WI, USA). The minimum inhibitory concentration (MIC) was defined as the lowest concentration that prevented visible bacterial growth.

### 3.8. Excision Wound Healing Study

#### 3.8.1. Model

The wound-healing study was conducted using male CD1 mice aged 2–3 months and weighing 30–40 g. The animals were obtained from the Animal Facility at Universidad Autónoma del Estado de Morelos (UAEM). All experimental procedures were previously approved by the institutional Animal Care and Use Committee of the Faculty of Medicine at UAEM (006/2022 approved by the Committee for the Care and Use of Laboratory Animals, FM-UAEM) and performed in accordance with the Technical Specifications for the Care and Use of Laboratory Animals (NOM-062-ZOO-1999, modified in 2001).

#### 3.8.2. Excision Wound Method

A total of 20 mice were randomly assigned to four groups (*n* = 5): a positive control (KitosCell^®^), a negative control (DMSO), and two experimental groups treated with T28-etOAc extract at 1 and 5 mg/mL. The animals were acclimatized for 7 days under standard laboratory conditions and housed individually with *ad libitum* access to food and water.

Anesthesia was induced via intraperitoneal injection of sodium pentobarbital (0.06 mg/g (Merck, Darmstadt, Germany, CAS 57-33.0)). The dorsal region was shaved, and a full-thickness excision wound (6 mm diameter) was created using a biopsy punch (day 0), as per the methodology described by López-Salazar et al., 2025 [86].

The extract, obtained in solid form, was dissolved in 1% (*v*/*v*) DMSO for topical application. A 10 μL volume of each treatment was applied directly to the wound area in only the first three days.

Wound-healing progression was documented via digital imaging using an AmScope camera (model Mu1000; USB 2.0, DC 5 V/250 mA, P/N: US901000A, L/N: US2111011; Ningbo, China) coupled to a VELAB stereo zoom microscope (model VE-S5; (CA 110/240 V, 50/60 Hz), equipped with WF 10X/20 mm eyepieces (Pharr, TX, USA)). Wound areas were measured using ImageJ software (version 2.4).

#### 3.8.3. Measurement of Wound Concentration

Wound contraction was evaluated on days 0, 1, 3, 6, 8, 10, and 13. The analysis was performed using normalized wound area, which was calculated as follows:

Normalized wound area = (A_n_ × 100)/(A_0_) (5)

Here, A_0_ is the wound area on day 0, and A_n_ is the wound area on day n.

The epithelization period was determined by recording the number of days required for complete eschar detachment, with no exposed wound surface remaining. The percentage of wound contraction was calculated using the following formula:

% wound contraction = (A_0_ − A_n_)/(A_0_) × 100 (6)

### 3.9. Statistical Analysis

The experiments were conducted using a completely randomized design with five independent replicates per treatment, and each experimental unit consisted of four explants. The response variable was the percentage of callus induction, expressed as mean ± standard error (SE). Data from leaf and node explants were analyzed separately using one-way analysis of variance (ANOVA), with treatment as a fixed factor. When significant differences were detected, means were compared using Tukey’s multiple comparison test at a significance level of *p* < 0.05.

Wound-healing results were analyzed using one-way ANOVA followed by Tukey’s post hoc test (*p* < 0.05). All statistical analyses were performed using Minitab statistical software (version 19.1.0). Graphs were generated using GraphPad Prism (version 9.3.0; GraphPad Software, San Diego, CA, USA).

## 4. Conclusions

This study demonstrates that callus cultures from *L. octovalvis* constitute a viable alternative for the controlled production of secondary metabolites with therapeutic relevance. The induction of friable calluses from leaf explants was achieved by combining 6-benzylaminopurine (4.44 µM) and 2,4-dichlorophenoxyacetic acid (0.45 µM), thereby establishing a stable, metabolically active cellular system. The extracts obtained showed distinct biological activities: the methanolic extract exhibited antimicrobial activity against methicillin-susceptible and methicillin-resistant *Staphylococcus aureus*, while the ethyl acetate extract promoted wound healing in a murine model by modulating key phases of the reparative process. Phytochemical analysis using GC-MS identified long-chain fatty acids and phytosterols, including *n*-hexadecanoic acid, oleic acid, stigmasterol, γ-sitosterol, and β-sitosterol, compounds that could explain the activity observed. Taken together, the results support *L. octovalvis* callus cultures’ potential as a sustainable biotechnological platform with which to produce secondary metabolites with antimicrobial and wound-healing applications. These findings facilitate the exploration of these cultures’ use in developing pharmaceutical and biomedical products with which to treat infections and promote tissue repair.

## Figures and Tables

**Table 1:** Effect of PGRs on callus induction in leaf and nodal explants of *L. octovalvis*.

PGRs	Concentration (µM)	Treatment	Explants	
			Leaf	Node
0	0	T0	0.00 ± 0.00 ^e^	0.00 ± 0.00 ^c^
BAP	0.44	T1	0.00 ± 0.00 ^e^	0.00 ± 0.00 ^c^
	2.22	T2	35.00 ± 6.08 ^bc^	0.00 ± 0.00 ^c^
	4.44	T3	0.00 ± 0.00 ^e^	0.00 ± 0.00 ^c^
KIN	0.46	T4	30.00 ± 4.99 ^cd^	0.00 ± 0.00 ^c^
	2.32	T5	0.00 ± 0.00 ^e^	0.00 ± 0.00 ^c^
	4.65	T6	0.00 ± 0.00 ^e^	0.00 ± 0.00 ^c^
NAA	0.54	T7	0.00 ± 0.00 ^e^	0.00 ± 0.00 ^c^
	2.69	T8	0.00 ± 0.00 ^e^	0.00 ± 0.00 ^c^
	5.37	T9	0.00 ± 0.00 ^e^	0.00 ± 0.00 ^c^
2,4-D	0.45	T10	0.00 ± 0.00 ^e^	0.00 ± 0.00 ^c^
	2.26	T11	0.00 ± 0.00 ^e^	0.00 ± 0.00 ^c^
	4.52	T12	0.00 ± 0.00 ^e^	0.00 ± 0.00 ^c^
BAP/NAA	0.44/0.54	T13	0.00 ± 0.00 ^e^	0.00 ± 0.00 ^c^
	0.44/2.69	T14	0.00 ± 0.00 ^e^	0.00 ± 0.00 ^c^
	0.44/5.37	T15	0.00 ± 0.00 ^e^	0.00 ± 0.00 ^c^
	2.22/0.54	T16	0.00 ± 0.00 ^e^	0.00 ± 0.00 ^c^
	2.22/2.69	T17	0.00 ± 0.00 ^e^	0.00 ± 0.00 ^c^
	2.22/5.37	T18	0.00 ± 0.00 ^e^	25.00 ± 0.00 ^a^
	4.44/0.54	T19	0.00 ± 0.00 ^e^	0.00 ± 0.00 ^c^
	4.44/2.69	T20	50.00 ± 0.00 ^b^	0.00 ± 0.00 ^c^
	4.44/5.37	T21	100.00 ± 0.00 ^a^	0.00 ± 0.00 ^c^
BAP/2,4-D	0.44/0.45	T22	87.50 ± 5.59 ^a^	0.00 ± 0.00 ^c^
	0.44/2.26	T23	30.00 ± 5.17 ^cd^	0.00 ± 0.00 ^c^
	0.44/4.52	T24	95.00 ± 5.17 ^a^	5.00 ± 0.00 ^b^
	2.22/0.45	T25	30.00 ± 5.17 ^cd^	0.00 ± 0.00 ^c^
	2.22/2.26	T26	95.00 ± 5.17 ^a^	0.00 ± 0.00 ^c^
	2.22/4.52	T27	35.00 ± 6.12 ^bc^	0.00 ± 0.00 ^c^
	4.44/0.45	T28	90.00 ± 5.17 ^a^	0.00 ± 0.00 ^c^
	4.44/2.26	T29	37.50 ± 5.59 ^bc^	0.00 ± 0.00 ^c^
	4.44/4.52	T30	41.00 ± 5.56 ^bc^	0.00 ± 0.00 ^c^
KIN/NAA	0.46/0.54	T31	0.00 ± 0.00 ^e^	0.00 ± 0.00 ^c^
	0.46/2.69	T32	0.00 ± 0.00 ^e^	0.00 ± 0.00 ^c^
	0.46/5.37	T33	0.00 ± 0.00 ^e^	0.00 ± 0.00 ^c^
	2.32/0.54	T34	0.00 ± 0.00 ^e^	0.00 ± 0.00 ^c^
	2.32/2.69	T35	0.00 ± 0.00 ^e^	0.00 ± 0.00 ^c^
	2.32/5.37	T36	0.00 ± 0.00 ^e^	25.00 ± 0.00 ^a^
	4.65/0.54	T37	0.00 ± 0.00 ^e^	0.00 ± 0.00 ^c^
	4.65/2.69	T38	0.00 ± 0.00 ^e^	0.00 ± 0.00 ^c^
	4.65/5.37	T39	0.00 ± 0.00 ^e^	0.00 ± 0.00 ^c^
KIN/2,4-D	0.46/0.45	T40	0.00 ± 0.00 ^e^	0.00 ± 0.00 ^c^
	0.46/2.26	T41	17.50 ± 3.06 ^d^	0.00 ± 0.00 ^c^
	0.46/4.52	T42	37.50 ± 5.59 ^bc^	0.00 ± 0.00 ^c^
	2.32/0.45	T43	37.50 ± 5.59 ^bc^	0.00 ± 0.00 ^c^
	2.32/2.26	T44	33.33 ± 4.56 ^bcd^	0.00 ± 0.00 ^c^
	2.32/4.52	T45	37.50 ± 5.59 ^bc^	0.00 ± 0.00 ^c^
	4.65/0.45	T46	0.00 ± 0.00 ^e^	0.00 ± 0.00 ^c^
	4.65/2.26	T47	0.00 ± 0.00 ^e^	0.00 ± 0.00 ^c^
	4.65/4.52	T48	0.00 ± 0.00 ^e^	0.00 ± 0.00 ^c^

Data recorded 40 days after inoculation. Means ± standard error (*n* = 5) followed by the same superscript letter in the column are statistically similar at the *p* < 0.05 level according to the Tukey multiple-range test. PGR: Plant growth regulator. BAP: 6-Benzylaminopurine. KIN: Kinetin. NAA: Naphthaleneacetic acid. 2,4-D: 2,4-Dichlorophenoxyacetic acid. *n* = 5.

**Table 2:** GC–MS profile and relative abundance of compounds in solvent extracts of callus cultures under T28 treatment.

\	Compound	Formula	Rt (min)	% Relative Abundance		
				Hex	EtOAc	MeOH
1	2,3-dihydro-3,5-dihydroxy-6-methyl-4H-pyran-4-one	C_6_H_8_O_4_	9.205	-	-	15.50
2	Cellobiose	C_12_H_22_O_11_	9.218	-	3.08	0.83
3	5-Hydroxymethylfurfural	C_6_H_6_O_3_	10.723	-	-	39.89
4	Methyl 6-oxoheptanoate	C_8_H_14_O_3_	12.043	-	0.28	-
5	Desulfosinigrin	C_10_H_17_NO_6_S	14.145	-	0.72	-
6	D-Mannose	C_6_H_12_O_6_	14.749	-	4.32	14.86
7	Isopropyl palmitate	C_19_H_38_O_2_	19.308	0.84	-	-
8	Palmitoleic acid	C_16_H_30_O_2_	20.267	0.74	-	-
9	*n*-Hexadecanoic acid	C_16_H_32_O_2_	18.881	1.11	11.74	5.56
10	Oleic acid	C_18_H_34_O_2_	21.889	2.85	4.87	2.09
11	7-Dehydrodiosgenin	C_27_H_40_O_3_	31.565	9.89	11.96	-
12	1-Heptatriacotanol	C_37_H_76_O	34.31	1.20	-	-
13	Stigmasterol	C_29_H_48_O	34.876	1.35	6.76	-
14	*γ*-Sitosterol	C_29_H_50_O	35.171	17.42	12.80	2.27
15	*β*-Sitosterol	C_29_H_50_O	36.262	-	0.59	-

Biomass from 42-day-old calli was used to obtain the extracts. Rt—retention time; Hex—hexane extract of T28 callus; EtOAc—ethyl acetate extract of T28 callus; MeOH—methanolic extract of T28 callus.

**Table 3:** Pharmacological activities with a Pa ≥ 0.5 related to the antimicrobial activity of compounds identified from *L. octovalvis* callus cultures.

Activity	Compounds
Antibacterial	5
Antifungal	11, 15 and 14
Antihelmintic (Nematodes)	9 and 10
Anti-infective	8, 9 and 10
Antiseptic	9
Antispirochetal	9
Antiprotozal	11
Antiviral	1, 9, 8, 10, 13, 15 and 14
Aspergillus nuclease S1 inhibitor	1
Chitinase inhibitor	1, 8 and 5
Dextranase inhibitor	1, 8 and 13
DNA polymerase I inhibitor	13
Endo-1,3(4)-β-glucanase inhibitor	1, 3 and 5
Fragilysin inhibitor	1, 3, 8 and 10
General pump inhibitor	11
Glucan 1,4-α-maltotriohydrolase inhibitor	3, 8
Glucan endo-1,3-β-D-glucosidase inhibitor	1, 3, 8, 13 and 14
Glucan endo-1,6-β-glucosidase inhibitor	1, 3 and 8
Histidine kinase inhibitor	1
IgA-specific metalloendopeptidase inhibitor	1, 3, 8 and 10
IgA-specific serine endopeptidase inhibitor	10
Lysostaphin inhibitor	1, 3 and 8
Muramoylpentapeptide transferase inhibitor	8
Mycothiol-S-conjugate amidase inhibitor	5
Omptin inhibitor	1, 3 and 8
Pediculicide	9
Peptidoglycan biosynthesis inhibitor	3
Peptidoglycan glycosyltransferase inhibitor	8, 13 and 14
Pseudolysin inhibitor	1, 3 and 8
RNA-directed RNA polymerase inhibitor	10
Signal peptidase I inhibitor	13
TPR proteinase inhibitor	10
Undecaprenyldiphospho-muramoylpentapeptide inhibitor	1

Note: Pa: Probability of Activity predicted by PASS Online; only activities with Pa ≥ 0.5 are shown.

**Table 4:** Pharmacological activity with a Pa ≥ 0.5 related to the wound-healing activity of compounds identified in *L. octovalvis* callus cultures.

Activity	Compounds
Anti-inflammatory	8, 9, 10, 11, 13, and 15
Angiogenesis stimulant	8, 9, and 10
Cytoprotectant	1, 8, 13, 14, and 15
Erythropoiesis stimulant	10 and 13
Fibroblast growth factor agonist	9, 8, and 10
HMOX1 expression enhancer	10 and 13
Leukopoiesis stimulant	10
Macrophage stimulant	10
Membrane integrity agonist	1, 3, and 8
Membrane permeability inhibitor	1 and 8
Mucomembranous protector	8, 14, and 15
Procollagen C-endopeptidase inhibitor	9
Procollagen N-endopeptidase inhibitor	8 and 9
Procollagen-proline dioxygenase inhibitor	9
Protein synthesis stimulant	13
Vasoprotector	1 and 9
Wound-healing agent	15 and 14

Note: Pa: Probability of Activity predicted by PASS Online; only activities with Pa ≥ 0.5 are shown.

**Table 5:** Minimum inhibitory concentration (MIC) of extracts from callus.

Treatment	MIC (µg/mL)			
	*S. aureus* ATCC 6538	MRSA ATCC 43300	*E. coli* ATCC 8739	*P. aeruginosa* Clinical Isolate
Gentamicin	≤0.78	12.5	<0.39	≤0.78
T28-Hex	>1000	>1000	>1000	>1000
T28-EtOAc	500	250	>1000	>1000
T28-MeOH	31.25	62.5	>1000	>1000

MRSA, methicillin-resistant *Staphylococcus aureus*; T28-Hex, hexane extract of T28 callus; T28-EtOAc, ethyl acetate extract of T28 callus; T28-MeOH, methanolic extract of T28 callus.

**Table 6:** Standardized residual wound area for each treatment per day.

Treatment	Day 1	Day 3	Day 6	Day 8	Day 10	Day 13
KitosCell^®^ (+)	43.06 ± 3.27 ^b^	22.76 ± 2.84 ^c^	19.67 ± 0.72 ^b^	8.99 ± 1.59 ^a^	5.37 ± 0.12 ^a^	0.00 ± 0 ^a^
DMSO (−)	89.17 ± 1.86 ^a^	79.42 ± 3.94 ^a^	43.84 ± 2.95 ^a^	7.87 ± 1.00 ^a^	0.56 ± 0.20 ^b^	0.32 ± 0.14 ^b^
1 mg/mL T28-EtOAc	48.37 ± 2.96 ^b^	35.75 ± 2.26 ^b^	19.29 ± 1.65 ^b^	1.86 ± 0.37 ^b^	0.35 ± 0.15 ^b^	0.00 ± 0 ^a^
5 mg/mL T28-EtOAc	80.79 ± 0.20 ^a^	72.23 ± 3.39 ^a^	26.52 ± 1.93 ^b^	8.42 ± 0.59 ^a^	1.68 ± 0.89 ^b^	0.00 ± 0 ^a^

Data are expressed as the mean ± standard error (*n* = 5). Different superscript letters indicate significant differences (*p* < 0.05). Positive control group (KitosCell); negative control group without treatment (1% DMSO); T28—ethyl acetate extract of T28 callus.

## Data Availability

The original contributions presented in this study are included in the article/supplementary material. Further inquiries can be directed to the corresponding author(s).

## Supplementary Materials

The following supporting information can be downloaded at: https://www.mdpi.com/article/10.3390/plants15172590/s1.
