# Supplementary material for: Callus Induction in Ludwigia octovalvis: Chemical Characterization and Potential Pharmacological Applications
Source: Plants (Basel). 2026 Aug 25;15(17):2590. doi: 10.3390/plants15172590 (PMC13567689; doi:10.3390/plants15172590)
Supplement: Supplementary file 1 [file plants-15-02590-s001.zip › plants-4407764-supplementary.pdf]

Supplementary Chromatograms

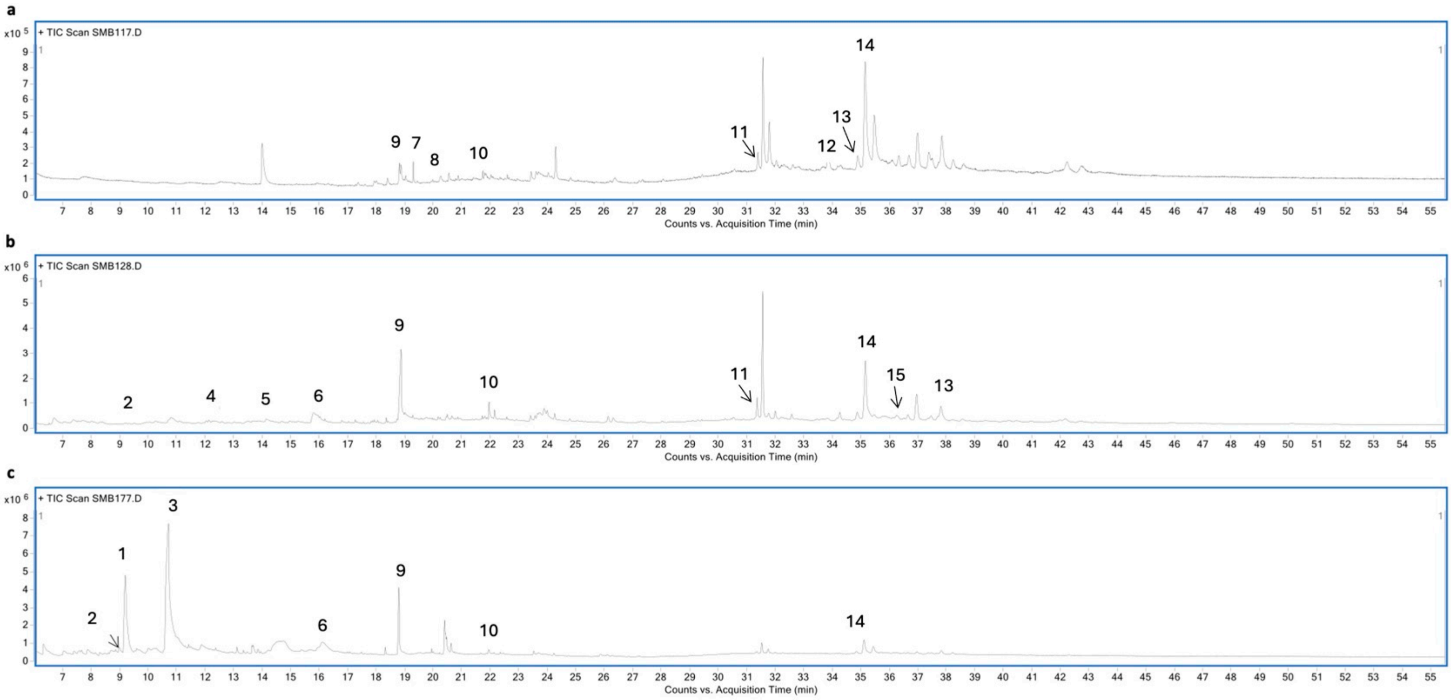

Figure S1. Chromatograms of T28 callus extracts obtained using different solvents: a) hexane, b) ethyl acetate, and c) methanol.
